# Supplementary material for: Plant–necrotroph co-transcriptome networks illuminate a metabolic battlefield
Source: eLife. 2019 May 13;8:e44279. doi: 10.7554/eLife.44279 (PMC6557632; doi:10.7554/eLife.44279)
Supplement: Transparent reporting form [file elife-44279-transrepform.docx]

***eLife’s* transparent reporting form**

We encourage authors to provide detailed information *within their submission* to facilitate the interpretation and replication of experiments. Authors can upload supporting documentation to indicate the use of appropriate reporting guidelines for health-related research (see [EQUATOR Network](http://www.equator-network.org/%20)), life science research (see the [BioSharing Information Resource](https://biosharing.org/)), or the [ARRIVE guidelines](http://www.plosbiology.org/article/info:doi/10.1371/journal.pbio.1000412) for reporting work involving animal research. Where applicable, authors should refer to any relevant reporting standards documents in this form.

If you have any questions, please consult our Journal Policies and/or contact us: [editorial@elifesciences.org](mailto:editorial@elifesciences.org).

**Sample-size estimation**

- You should state whether an appropriate sample size was computed when the study was being designed
- You should state the statistical method of sample size computation and any required assumptions
- If no explicit power analysis was used, you should describe how you decided what sample (replicate) size (number) to use

Please outline where this information can be found within the submission (e.g., sections or figure legends), or explain why this information doesn’t apply to your submission:

The description for samples listed in Methods Section (Page31 to Page39). Our study utilized a collection of 96 isolates of *B. cinerea* to infect on three Arabidopsis genotypes in a random design with 6-fold replication across the two independent experiments. For statistical analysis of pathogen gene expression, we first filtered genes with either more than 30 gene counts in one isolate or 300 gene counts across 96 isolates. We normalized B. cinerea gene counts data set using the trimmed mean of M-values method (TMM) then ran the generalized linear model (GLM) with a negative binomial link function. The least square means of pathogen transcripts were used for downstream analysis.

**Replicates**

- You should report how often each experiment was performed
- You should include a definition of biological versus technical replication
- The data obtained should be provided and sufficient information should be provided to indicate the number of independent biological and/or technical replicates
- If you encountered any outliers, you should describe how these were handled
- Criteria for exclusion/inclusion of data should be clearly stated
- High-throughput sequence data should be uploaded before submission, with a private link for reviewers provided (these are available from both GEO and ArrayExpress)

Please outline where this information can be found within the submission (e.g., sections or figure legends), or explain why this information doesn’t apply to your submission:

Experimental design and number of biological replicates reported in Methods Section (page31 to page39). Two independent randomized complete block-designed experiments were conducted with 6-fold replication across the two experiments. A total of twelve infected leaves per isolate/genotype pair were generated, which resulted in a total of 1,052 RNA-Seq libraries and 2,104 measures for lesion size and chemical analysis. The sequencing data in this study are available in the following database: Bioproject PRJNA473829 (<https://www.ncbi.nlm.nih.gov/bioproject/?term=PRJNA473829>).

**Statistical reporting**

- Statistical analysis methods should be described and justified
- Raw data should be presented in figures whenever informative to do so (typically when N per group is less than 10)
- For each experiment, you should identify the statistical tests used, exact values of N, definitions of center, methods of multiple test correction, and dispersion and precision measures (e.g., mean, median, SD, SEM, confidence intervals; and, for the major substantive results, a measure of effect size (e.g., Pearson's r, Cohen's d)
- Report exact p-values wherever possible alongside the summary statistics and 95% confidence intervals. These should be reported for all key questions and not only when the p-value is less than 0.05.

Please outline where this information can be found within the submission (e.g., sections or figure legends), or explain why this information doesn’t apply to your submission:

Statistical methods and models are described in detail throughout the main text and methods section. All statistical tests are shown in all figures.

(For large datasets, or papers with a very large number of statistical tests, you may upload a single table file with tests, Ns, etc., with reference to sections in the manuscript.)

**Group allocation**

- Indicate how samples were allocated into experimental groups (in the case of clinical studies, please specify allocation to treatment method); if randomization was used, please also state if restricted randomization was applied
- Indicate if masking was used during group allocation, data collection and/or data analysis

Please outline where this information can be found within the submission (e.g., sections or figure legends), or explain why this information doesn’t apply to your submission:

The fungi isolates, culture methods, and plant growth conditions are reported in the Methods Section (Page31 to Page33).

**Additional data files (“source data”)**

- We encourage you to upload relevant additional data files, such as numerical data that are represented as a graph in a figure, or as a summary table
- Where provided, these should be in the most useful format, and they can be uploaded as “Source data” files linked to a main figure or table
- Include model definition files including the full list of parameters used
- Include code used for data analysis (e.g., R, MatLab)
- Avoid stating that data files are “available upon request”

Please indicate the figures or tables for which source data files have been provided:

All the relevant data have been provided in Supplementary files. The code used for data analysis is listed in the Data Availability (Page39).
